# Aging Affects Lower Limb Joint Moments and Muscle Responses to a Split-Belt Treadmill Perturbation

**DOI:** 10.3389/fspor.2021.683039

**Published:** 2021-07-19

**Authors:** Dongyual Yoo, Junmo An, Kap-Ho Seo, Beom-Chan Lee

**Affiliations:** ^1^Department of Health and Human Performance, Center for Neuromotor and Biomechanics Research, University of Houston, Houston, TX, United States; ^2^Korea Institute of Robotics and Technology Convergence, Pohang, South Korea; ^3^Department of Mechanical Engineering, Pohang University of Science and Technology, Pohang, South Korea; ^4^Michael E. DeBakey Veterans Affairs Medical Center, Houston, TX, United States

**Keywords:** falls, split-belt treadmill perturbation, aging, joint moments, muscle responses

## Abstract

Age-related changes cause more fall-related injuries and impede the recoveries by older adults compared to younger adults. This study assessed the lower limb joint moments and muscle responses to split-belt treadmill perturbations in two groups (14 healthy young group [23.36 ± 2.90 years] and 14 healthy older group [70.93 ± 4.36 years]) who performed two trials of unexpected split-belt treadmill perturbations while walking on a programmable split-belt treadmill. A motion capture system quantified the lower limb joint moments, and a wireless electromyography system recorded the lower limb muscle responses. The compensatory limb's (i.e., the tripped limb's contralateral side) joint moments and muscle responses were computed during the pre-perturbation period (the five gait cycles before the onset of a split-belt treadmill perturbation) and the recovery period (from the split-belt treadmill perturbation to the baseline gait relying on the ground reaction forces' profile). Joint moments were assessed by maximum joint moments, and muscle responses were quantified by the normalization (%) and co-contraction index (CCI). Joint moments and muscle responses of the compensatory limb during the recovery period were significantly higher for the YG than the OG, and joint moments (e.g., knee flexion and extension and hip flexion moments) and muscle responses during the recovery period were higher compared to the pre-perturbation period for both groups. For CCI, the older group showed significantly higher co-contraction for biceps femoris/rectus femoris muscles than the young group during the recovery period. For both groups, co-contraction for biceps femoris/rectus femoris muscles was higher during the pre-perturbation period than the recovery period. The study confirmed that older adults compensated for muscle weakness by using lower joint moments and muscle activations and increasing muscle co-contractions to recover balance after split-belt treadmill perturbations. A better understanding of the recovery mechanisms of older adults who train on fall-inducing systems could improve therapeutic regimens.

## Introduction

Falls are the leading cause of injury and death in young and older adults (Berg et al., 1997; Timsina et al., 2017). Falls affect quality of life due to fall-related physical injuries (fractures) and loss of confidence and fear of falling while engaging in daily activities (Tinetti et al., 1988; Salkeld et al., 2000; Gallagher et al., 2001). Falls have occurred during a variety of activities (e.g., walking, running, playing sports, going up/down the stairs, moving between sitting and standing positions, etc.) in young and older adults (Timsina et al., 2017), and walking and sports/exercise activities are the first and second fall-related activities throughout all ages, respectively (Talbot et al., 2005). Considering that walking is an important activity in daily activities (Lawton and Brody, 1969; Gill et al., 1997), unexpected gait perturbations, known as trips and slips, are the major causes of falls in young adults and the community-dwelling elderly (Berg et al., 1997; Heijnen and Rietdyk, 2016; Timsina et al., 2017). For sports activities, a concussion is one of the major sports-related injuries (Hutchison et al., 2015; Kendall et al., 2020). For example, in ice hockey and football sports, a fall to the ice or the ground and a trip are the causes of concussions (Hutchison et al., 2015; Kendall et al., 2020). Concussion results in impaired emotional, neurocognitive, and physical functioning (Conder and Conder, 2015).

Age-related cognitive and visual impairments, changes in neuromuscular mechanisms, and declining muscle strength increase the probability of tripping and falls in older adults (Pijnappels et al., 2008; Bento et al., 2010). Given that lower limbs contribute to balance recovery by creating a new base of support and generating joint moments to control balance stability after a gait perturbation (Wang et al., 2019, 2020; Yoo et al., 2019), reduced lower limb muscle strength caused by aging can be an important fall predictor (Horlings et al., 2008; Pijnappels et al., 2008). Decreased lower limb muscle strength is a limiting factor in preventing falls in older adults (Pijnappels et al., 2008). A previous study indicated that older adults with relatively weaker lower limb muscle strength showed a slower recovery process and higher fall rates after slip perturbations induced by a vinyl tile surface coated with oil compared to young adults (Lockhart et al., 2005). One study found that young adults used higher ankle and knee joint moments to recover balance after slip perturbations induced by a slippery mixture compared to older adults (Liu and Lockhart, 2009), while another found that young adults used higher knee and hip joint moments to avoid an obstacle after trip perturbations induced by the external obstacle compared to older adults (McFadyen and Prince, 2002).

Changes in the neuromuscular system such as decreases in motor unit firing rate and fewer motor units, affect older adults' muscle responses after losing balance (e.g., reduced muscle activation and increased co-contraction of agonist and antagonist muscles) (Tang and Woollacott, 1998; Okada et al., 2001; Lockhart and Kim, 2006; Chambers and Cham, 2007; Pijnappels et al., 2008; Bento et al., 2010; Watanabe et al., 2018). As muscle responses correlate to muscle strengths and joint moments (Buchanan et al., 2005; Watanabe et al., 2018), electromyography (EMG) has been analyzed to understand different muscle responses to gait perturbations for young and older adults (Tang and Woollacott, 1998; Lockhart and Kim, 2006). Older adults showed lower activation rates, smaller amplitudes, and longer onset latencies of lower limb muscles compared to young adults after slip perturbations induced by a moveable platform or a vinyl tile surface coated with a soap and water mixture (Tang and Woollacott, 1998; Lockhart and Kim, 2006).

When older adults confront balance challenges, they increase joint stiffness through muscle co-contraction to compensate for muscle weakness, which is a compensatory strategy to recover balance stability by decreasing the degree of freedom of the body segment's movement (Nagai et al., 2011; Nelson-Wong et al., 2012; Schinkel-Ivy and Duncan, 2018). Increased co-contraction of the gastrocnemius (GAS) and tibialis anterior (TA) muscles was found in older adults after balance perturbations induced by a moving platform (Okada et al., 2001). Older adults also showed higher co-contraction and a longer co-contraction duration at knee and hip muscles after slip perturbations induced by a moveable platform or a contaminated floor (Tang and Woollacott, 1998; Chambers and Cham, 2007).

Gait perturbation systems with external mechanisms have practical limitations because they require enough physical space for overground walking, have fixed locations, etc. (Schillings et al., 1996; Pavol et al., 1999; Cham and Redfern, 2002; Okubo et al., 2018). One alternative is a programmable treadmill with a single belt or dual belts (Sessoms et al., 2014; Mueller et al., 2016). Recently, we developed a fall-inducing system incorporating a split-belt treadmill (Lee et al., 2017a,b; Lee et al., 2019, 2020; Yoo et al., 2019). In our previous studies (Lee et al., 2017a,b, 2019, 2020; Yoo et al., 2019), we confirmed kinematic changes (i.e., increased maximum trunk flexion angle, maximum trunk flexion velocity, and maximum center of mass (COM) velocity) following a split-belt treadmill perturbation induced by instantaneously stopping one belt of our fall-inducing system. These results were similar to the results of previous studies demonstrating increased maximum trunk flexion angle, maximum trunk angular velocity, and COM position in the anterior direction after trip perturbations induced by mechanical obstacles (Pavol et al., 2001; Bieryla et al., 2007).

Although trip perturbations contribute to more fall-related injuries than slip perturbations in older adults (Timsina et al., 2017), and trip and slip perturbations result in different body responses (e.g., the forward and backward loss of balance for trip and slip perturbations, respectively) (Grabiner et al., 1993; Pavol et al., 2001; Pai et al., 2010), most studies have investigated the differences in lower limb joint moments and muscle responses to slip perturbations, rather than trip perturbations, by young and older adults. Therefore, this study assessed lower limb joint moments and muscle responses to split-belt treadmill perturbations in young and older adults. We hypothesized that 1) young adults would show higher lower limb joint moments and muscle activations after split-belt treadmill perturbations and 2) older adults would show higher muscle co-contractions after split-belt treadmill perturbations.

## Methods

### Participants

Based on the results of our previous studies (Lee et al., 2017a,b, 2019, 2020; Yoo et al., 2019) and our pilot study, the power analysis indicated a minimum of 20 participants, with an effect size (*f*) = 0.67 (large effect size) (Cohen, 1992), power (*1-*β) = 0.80, and α = 0.05. Fourteen healthy young adults [7 females and 7 males; Young Group (YG)] and 14 healthy older adults [10 females and 4 males; Older Group (OG)] participated as shown in Table 1. No participants had a major operation in the previous 6 months, musculoskeletal dysfunctions, or neurological and peripheral sensory diseases. All participants scored 26 or more in the Montreal Cognitive Assessment (MOCA), representing normal cognitive ability. All participants read and signed a consent form prior to the study, which was approved by the Institutional Review Boards of the University of Houston.

**Table 1:** Statistical analysis results of the demographic characteristics, walking speeds, and MOCA of the participants (*n* = 28) for young group (YG) and older group (OG).

	**YG (*n* = 14)**	**OG (*n* = 14)**	***p* value**
Gender (male, %)	Male, 50%	Male, 29%	–
Age (years)	23.36, 2.90	70.93, 4.36	<0.0001***
Weight (kg)	70.35, 14.20	72.43, 12.87	0.688
Height (cm)	172.11, 10.59	167.56, 7.08	0.192
BMI (kg/m^2^)	23.67, 3.75	25.85, 4.59	0.178
Walking speed (m/s)	0.86, 0.06	0.63, 0.14	<0.0001***
MOCA	28.86, 1.10	28.07, 1.07	0.067

BMI, body mass index; MOCA, montreal cognitive assessment (*** p < 0.0001).

### Experimental Procedures

**Figure 1:**
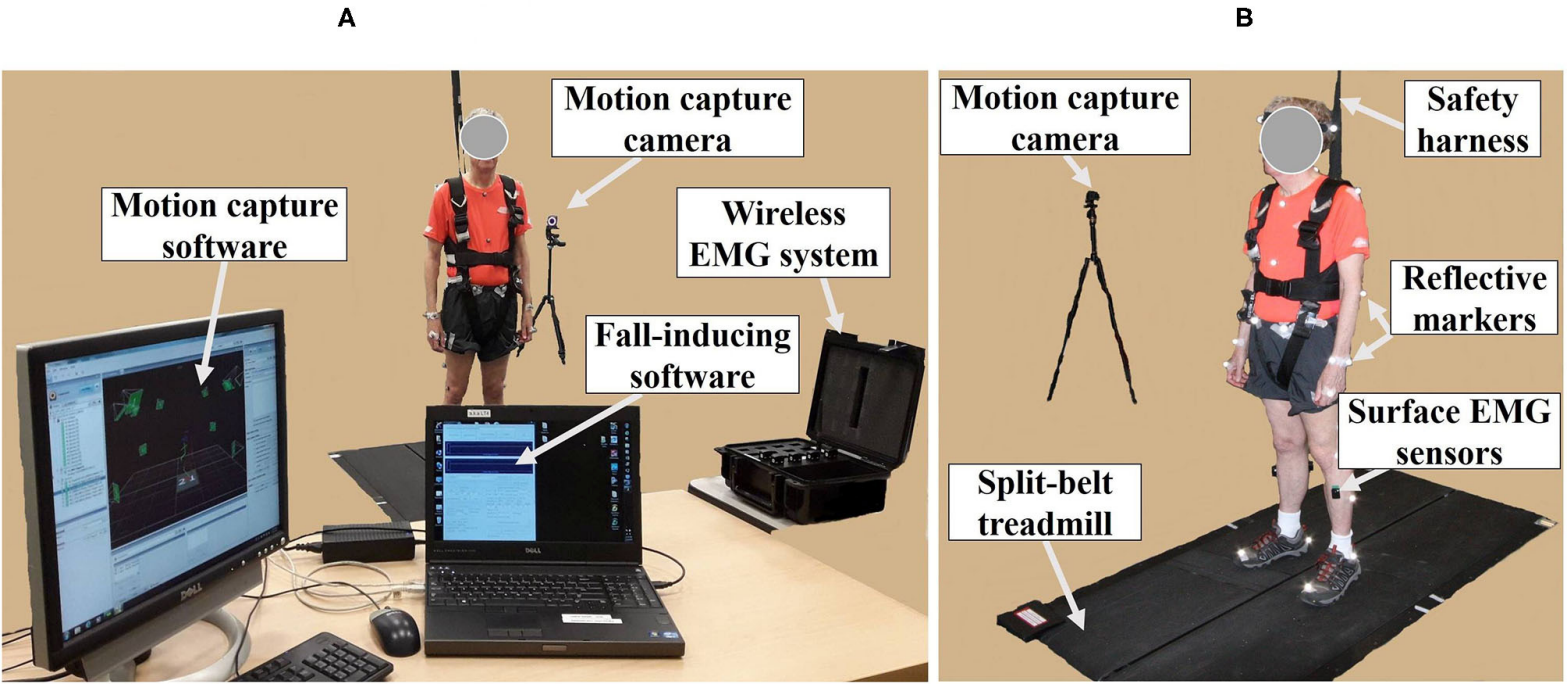
Experimental apparatus. **(A)** Motion capture system; wireless electromyography (EMG) system; fall-inducing system. **(B)** Reflective markers and surface EMG sensors placed on body landmarks; split-belt treadmill; safety harness; motion capture camera.

The fall-inducing system consisted of one load cell (LC101-250, Omega Engineering Inc., CT, USA), a programmable split-belt treadmill embedded with two force plates underneath (Bertec Corporation, Columbus, OH, USA), and a VICON motion capture system consisting of 35 reflective passive markers and 12 near-infrared cameras (Vicon Motion Systems Ltd., Oxford, UK), and custom software as shown in Figure 1. A wireless EMG system (Trigno ™IM, Delsys Inc., Natick, MA, USA) was used to measure muscle responses.

The Nexus 1.8 software synchronized with the EMG system and the custom software sampled the EMG signals at 2000 Hz and the marker positions and the ground reaction forces (GRFs) at 100 Hz, respectively [see the detailed algorithm information in (Lee et al., 2017a); see the custom software information in (Lee et al., 2017a, 2019; Yoo et al., 2019)]. The custom software generated split-belt treadmill perturbations (at the foot level) by stopping the treadmill's left belt within 100 ms at a rate of 10 m/s^2^ at 10% of the gait cycle (approximately the loading phase) determined by GRFs (Lee et al., 2017a, 2020; Yoo et al., 2019). After the non-tripped foot's first heel strike (i.e., the first heel strike of the right foot), the stationary treadmill belt returned to the pre-perturbation speed within 100 ms at a rate of 10 m/s^2^ (Lee et al., 2017a, 2020; Yoo et al., 2019). If the peak loading force measured by the load cell exceeded 30% of a participant's body weight, a trial was considered a fall incident (Bhatt et al., 2013; Okubo et al., 2018).

The 35 reflective passive markers were attached bilaterally on the body and the 10 wireless surface EMG sensors were attached to biceps femoris (BF), rectus femoris (RF), TA, and GAS (lateralis and medialis) muscles as shown in Figure 1B. All participants wore a safety harness and selected their own comfortable walking speed (0.86 ± 0.06 m/s for YG and 0.63 ± 0.14 m/s for OG) by increasing or decreasing the treadmill's speed until they felt comfortable as shown in Table 1. No participants received instructions (e.g., how to respond, recover, etc.) and there were no practice trials.

Since a previous study indicated that motor adaptation to trip perturbations occurred from the third trial compared to the first trial, all participants performed 2 consecutive split-belt treadmill perturbation trials (Wang et al., 2012) consisting of standing (15 s standing), pre-perturbation (steady walking at the self-selected walking speed with 31 to 40 gait cycles), and recovery (steady walking at the self-selected walking speed after the split-belt treadmill perturbation) periods. There was a 20 s rest period between the trials. There were no marker or system malfunctions.

The fall-inducing treadmill randomly induced a split-belt treadmill perturbation between the 31st and 40th steps at the left foot based on a study indicating that the number and percentages of recovery and fall were not affected by the side of the tripped foot (Pavol et al., 1999). Each trial ended after 15 steps from the split-belt treadmill perturbation to provide adequate recovery for the OG, since healthy young adults needed nearly 5 s for recovery (Lee et al., 2017a).

### Data Processing

Using the Nexus 1.8 software, the full body Plug-in-Gait model filtered the 35 reflective passive marker positions by a sixth-order Butterworth filter with a cut-off frequency of 6 Hz (Yoo et al., 2019) and computed the ankle, knee, and hip moments in the sagittal plane based on the filtered marker positions. The Plug-in-Gait model is a standard and reliable tool in biomechanics research for analyzing joint moments, especially for the sagittal plane (Kadaba et al., 1989). Previous studies found that the whole-body movement predominated in the sagittal plane after trip perturbations (Lee et al., 2017a, 2019). The low-pass filter was applied to the GRFs by a second-order Butterworth filter with a cut-off frequency of 10 Hz using MATLAB (The MathWorks, Natick, MA, USA) (Lee et al., 2019). A band pass filter was applied to the EMG signals by a fifth-order Butterworth filter with a low cut-off frequency of 20 Hz and a high cut-off frequency of 300 Hz (Lee et al., 2019).

Since the compensatory limb's stepping response (the contralateral side of tripped limb) is the general response to gait perturbations (Jensen et al., 2001; Maki and McIlroy, 2006; Yoo et al., 2019), joint moments (i.e., maximum right ankle dorsiflexion and plantarflexion, knee flexion and extension, and hip flexion and extension moments) and EMG signals (right BF, RF, TA, and GAS muscles) of the compensatory limb were analyzed. Since our previous study found no significant difference between EMG signals from the medial and lateral GAS before and after split-belt treadmill perturbations (Lee et al., 2019), the EMG signals from the medial and lateral GAS were averaged and analyzed. The joint moments and EMG signals were computed for the standing, pre-perturbation, and recovery periods. The recovery period was defined as the period from the instant of split-belt treadmill perturbation to return to baseline gait depending on the GRF's profile (i.e., when a correlation coefficient attained 95%). To compute the recovery period, each of the 5 gait cycle's GRFs before the split-belt treadmill perturbation was normalized (i.e., the GRFs ranged from 0 to 100% corresponding to the gait cycle) and then averaged. Each gait cycle's GRFs after the split-belt treadmill perturbation was also normalized and compared to the averaged GRFs before the split-belt treadmill perturbation with Pearson's correlation coefficient.

Consistent with our previous kinematic analysis (Lee et al., 2019), joint moments of pre-perturbation and recovery periods were normalized to the averaged joint moments of the standing period to remove baseline joint moments (i.e., averaged joint moments while standing) from joint moments during pre-perturbation and recovery periods. For EMG analysis, the filtered EMG signals were rectified and enveloped by the root-mean-square (RMS) using a 20 ms moving window (Begalle et al., 2012). EMG signals were normalized based on the maximum value during normal gait (Heiden et al., 2006; Qu et al., 2012). The EMG signals of 5 normal gait cycles during the pre-perturbation period were averaged. Next, the maximum value and the pre- and post-values from the maximum on the averaged signals were averaged for normalization and defined as a normalization value. For normalization, the mean of averaged EMG signals during the pre-perturbation period and the mean of EMG signals during the recovery period were divided by the normalization value and multiplied by 100, respectively.

$$\begin{array}{l} {\frac{Mean\;of\;averaged\;EMG\;signals\;during\;pre-perturbation\;period\;}{normalization\;value}}^{*}100\%\\ {\frac{Mean\;of\;EMG\;signals\;during\;recovery\;period}{normalization\;value}}^{*}100\% \end{array}$$

**Figure 2:**
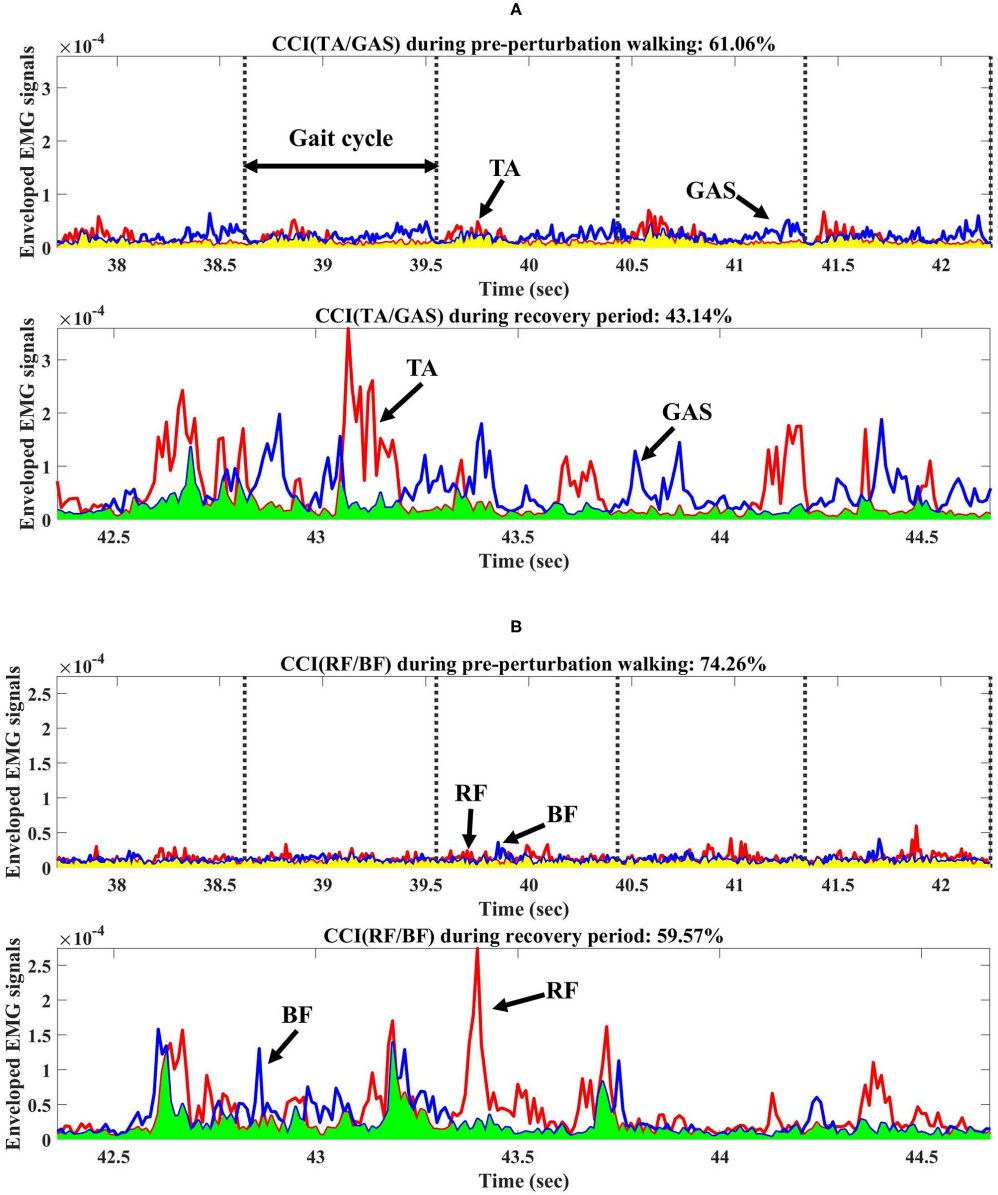
Co-contraction index (CCI) from one participant during pre-perturbation and recovery periods. **(A)** CCI of TA/GAS during pre-perturbation and recovery periods. **(B)** CCI of RF/BF during pre-perturbation and recovery periods. TA, GAS, RF, and BF indicate tibialis anterior, gastrocnemius, rectus femoris, and biceps femoris, respectively. Yellow shaded area indicates the area under the lower signals at any point between two muscle activity signals during pre-perturbation period used for CCI calculations. Light green shaded area indicates the area under the lower signals at any point between two muscle activity signals during recovery period.

CCI (i.e., between TA and GAS (the average of the medial and lateral GAS) and BF and RF, respectively) was computed for the pre-perturbation and recovery periods, respectively, as shown in Figure 2, based on Falconer and Winter (Falconer and Winter, 1985) as:

$$\begin{array}{l} CCI={\frac{2{\text{I}}_{ant}}{{\text{I}}_{total}}}^{*}100\%, \end{array}$$

where I_ant_ is the area of the lower signals at any point between two muscle activity signals and I_total_ is the sum of the area of the lower signals and the area of higher signals at any point between two muscle activity signals.

### Statistical Analysis

Statistical analysis was performed by SPSS (IBM Corp., Armonk, NY, USA) for join moments, normalized muscle activations, and CCI. Levene's test and the Shapiro-Wilk test, respectively, confirmed the homogeneity of variances and normal distributions of the outcome measures. An independent *t*-test was used to compare the YG and OG demographic characteristics, self-selected walking speeds, and MOCA. Two-way analysis of variance (ANOVA) was performed for six join moments (maximum right ankle dorsiflexion and plantarflexion, knee flexion and extension, and hip flexion and extension moments), four normalized muscle activations (EMG signals for right BF, RF, TA, and GAS muscles), and two CCI (CCI between TA and GAS and CCI between BF and RF) to evaluate the main effects of the period (pre-perturbation and recovery periods), groups, and their interactions. An *F* test was used to identify the main effects and the interaction effects, and *post-hoc* analysis (Šídák method) was conducted to confirm the influence of any factors on the main and interaction effects. The significance levels for statistical analyses were set at *p* < 0.05.

## Results

### Demographic Characteristics and Recovery Steps

The results of the independent *t*-test showed that age and walking speed differed significantly, whereas there were no significant differences for weight, height, BMI, and MOCA between the two groups as reported in Table 1. Following the split-belt treadmill perturbations, the YG and OG returned to their normal walking in 1.93 ± 1.02 steps and 2.43 ± 1.23 steps, respectively.

### Joint Moments

**Figure 3:**
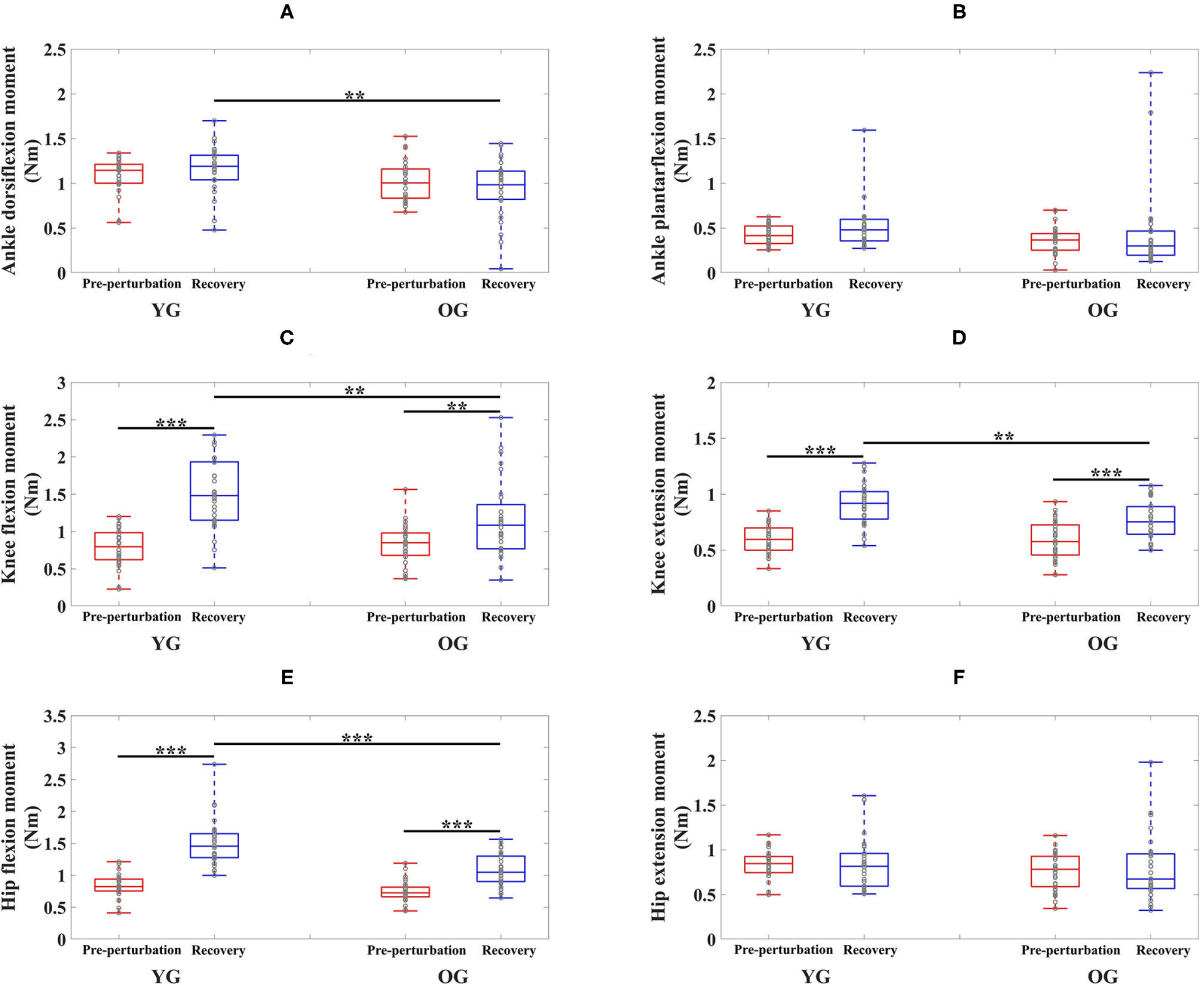
Box plots of joint moments as a function of group (YG and OG) and period across all participants (**p < 0.01 and ***p < 0.0001). **(A)** Ankle dorsiflexion moment. **(B)** Ankle plantarflexion moment. **(C)** Knee flexion moment. **(D)** Knee extension moment. **(E)** Hip flexion moment. **(F)** Hip extension moment. YG and OG indicate young group and older group, respectively. Gray circles superimposed on box plots indicate individual data points from all participants.

The results of the two-way ANOVA showed a significant main effect of the group for ankle dorsiflexion moments and no significant main and interaction effects for ankle plantarflexion moment as reported in Table 2. *Post-hoc* analysis indicated that the YG showed significantly higher ankle dorsiflexion moments during the recovery period than the OG (*p* = 0.002) as shown in Figure 3A. However, ankle plantarflexion moments were not significantly different within and between groups as shown in Figure 3B.

**Table 2:** Statistical analysis results of joint moments for group (G), period (P), and interaction (G × P) (^*^p < 0.05, ^**^p < 0.01, and ^***^p < 0.0001).

**Joint moments**	**Effects**	**DF**	***F* value**	***p* value**
Dorsiflexion moment	G	1, 108	9.448	0.003**
	P	1, 108	0.011	0.916
	G × P	1, 108	2.296	0.133
Plantarflexion moment	G	1, 108	1.860	0.175
	P	1, 108	2.074	0.153
	G × P	1, 108	0.003	0.953
Knee flexion moment	G	1, 108	3.886	0.051
	P	1, 108	49.796	<0.0001***
	G × P	1, 108	6.411	0.013*
Knee extension moment	G	1, 108	5.813	0.018*
	P	1, 108	60.595	<0.0001***
	G × P	1, 108	4.241	0.042*
Hip flexion moment	G	1, 108	27.684	<0.0001***
	P	1, 108	107.505	<0.0001***
	G × P	1, 108	11.955	0.001**
Hip extension moment	G	1, 108	1.274	0.262
	P	1, 108	0.118	0.732
	G × P	1, 108	0.188	0.665

*The period indicates the pre-perturbation period and recovery period*.

For knee joint moments, the results of the two-way ANOVA showed a significant main effect of the period and the interaction effect for knee flexion moments, and significant main effects of the group and period, and the interaction effect for knee extension moments, respectively as shown in Table 2. *Post-hoc* analysis indicated that the YG showed significantly higher knee flexion moments (*p* = 0.002) and extension moments (*p* = 0.002) during the recovery period than the OG as shown in Figures 3C,D. Both groups showed significantly increased knee flexion moments (YG: *p* < 0.0001 and OG: *p* = 0.002) and knee extension moments (YG: *p* < 0.0001 and OG: *p* < 0.0001) during the recovery period compared to the pre-perturbation period.

For hip joint moments, the two-way ANOVA indicated significant main effects of the group and period, and the interaction effect for hip flexion moment, and no significant main and interaction effects for hip extension moment as shown in Table 2. *Post-hoc* analysis indicated that the YG showed significantly higher hip flexion moments during the recovery period compared to the OG (*p* < 0.0001) as shown in Figure 3E. Both groups showed significantly increased hip flexion moments during the recovery period compared to the pre-perturbation period (YG: *p* < 0.0001 and OG: *p* < 0.0001). However, hip flexion moments were not significantly different within and between groups as shown in Figure 3F.

### Muscle Responses

**Figure 4:**
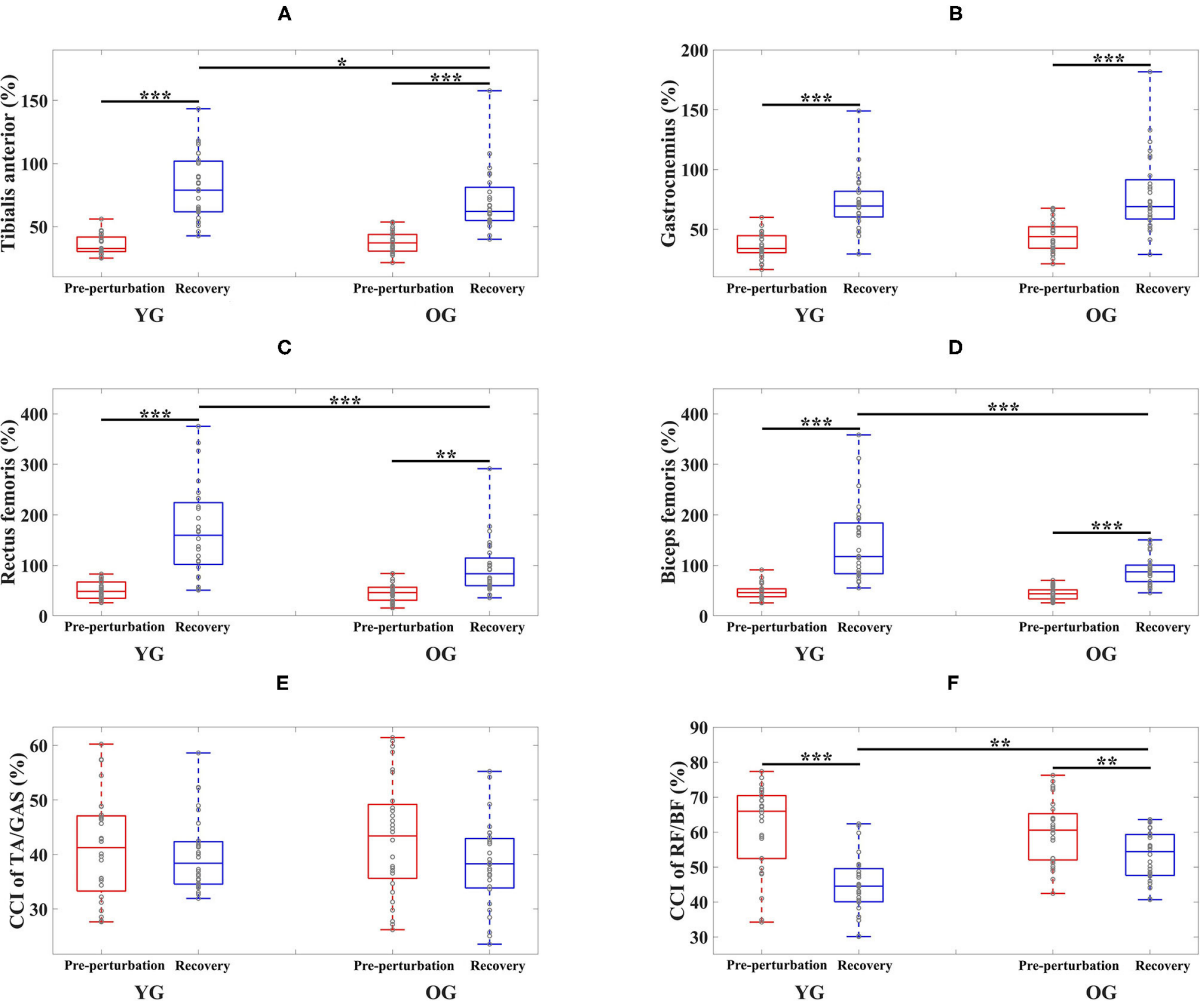
Box plots of normalized muscle activations and co-contraction index (CCI) as a function of group (YG and OG) and period across all participants (*p < 0.05, **p < 0.01, and ***p < 0.0001). **(A)** Tibialis anterior. **(B)** Gastrocnemius. **(C)** Rectus femoris. **(D)** Biceps femoris. **(E)** CCI of TA/GAS. **(F)** CCI of RF/BF. YG and OG indicate young group and older group, respectively. TA, GAS, RF, and BF indicate tibialis anterior, gastrocnemius, rectus femoris, and biceps femoris, respectively. Gray circles superimposed on box plots indicate individual data points from all participants.

The results of the two-way ANOVA indicated a significant main effect of the period for the TA and GAS muscles as shown in Table 3. *Post-hoc* analysis indicated that the YG showed significantly higher TA muscle activation during the recovery period than the OG (*p* = 0.034). Both groups showed significantly higher muscle activations during the recovery period compared to the pre-perturbation period (YG: *p* < 0.0001 and OG: *p* < 0.0001) as shown in Figure 4A. Both groups showed significantly higher muscle activations for the GAS muscle during the recovery period compared to the pre-perturbation period (YG: *p* < 0.0001 and OG: *p* < 0.0001) as shown in Figure 4B.

**Table 3:** Statistical analysis results of normalized muscle activation for group (G), period (P), and interaction (G×P) (^**^p < 0.01 and ^***^p < 0.0001).

**Normalization**	**Effects**	**DF**	***F* value**	***p* value**
Tibialis anterior	G	1, 108	1.709	0.194
	P	1, 108	120.132	<0.0001***
	G × P	1, 108	2.969	0.088
Gastrocnemius	G	1, 108	3.224	0.075
	P	1, 108	75.514	<0.0001***
	G × P	1, 108	0.021	0.885
Rectus femoris	G	1, 108	13.456	<0.0001***
	P	1, 108	65.802	<0.0001***
	G × P	1, 108	10.211	0.002**
Biceps femoris	G	1, 108	12.730	0.001**
	P	1, 108	81.904	<0.0001***
	G × P	1, 108	9.659	0.002**

*The period indicates the pre-perturbation period and recovery period*.

The results of the two-way ANOVA indicated significant main effects of the group and period and the interaction effect for the RF and BF muscles as reported in Table 3. *Post-hoc* analysis indicated that the YG showed significantly higher muscle activations during the recovery period than the OG (RF (*p* < 0.0001) and BF (*p* < 0.0001)) as shown in Figures 4C,D. Both groups showed significantly higher RF (YG: *p* < 0.0001 and OG: *p* = 0.001) and BF (YG: *p* < 0.0001 and OG: *p* < 0.0001) muscle activations during the recovery period compared to the pre-perturbation period.

The results of the two-way ANOVA indicated a significant main effect of the period [*F*_(1, 108)_ = 44.170, *p* < 0.0001] and the interaction effect [*F*_(1, 108)_ = 7.725, *p* = 0.006] for the CCI of BF/RF (Figure 4F), and an insignificant main effect and interaction effect for the CCI of TA/GAS (Figure 4E). *Post hoc* analysis indicated that the OG showed significantly higher CCI of BF/RF during the recovery period (*p* = 0.001) than the YG. Both groups showed higher CCI of BF/RF during the pre-perturbation period compared to the recovery period (YG: *p* < 0.0001 and OG: *p* = 0.007).

## Discussion

This study investigated young and older adults' joint moments and muscle responses of the compensatory limb to split-belt treadmill perturbations. Both groups showed overall increased joint moments and muscle activations during the recovery period compared to the pre-perturbation period. The YG showed higher joint moments and muscle activations during the recovery period than the OG, whereas the OG showed higher muscle co-contractions of BF/RF during the recovery period.

This study confirmed previous findings of increased joint moments of the compensatory limb (e.g., increased peak ankle, knee, and hip joint moments) by older adults to control the body's forward rotation and to gain time and clearance for repositioning the tripped foot after trip perturbations induced by an external obstacle (Pijnappels et al., 2004; 2005). This study, however, indicated insignificant increases in plantarflexion and hip extension moments after split-belt treadmill perturbations induced by stopping one belt of a split-belt treadmill, which contradicted a previous study indicating increased plantarflexion and hip extension moments after trip perturbations induced by an external obstacle (Pijnappels et al., 2005). Since a small sample size affect statistical power (Suresh and Chandrashekara, 2012), this result could not be generalized. However, we speculate that the plantarflexion and hip extension moments contributing to push-off for foot clearance of the tripped limb were unnecessary after split-belt treadmill perturbations (Pijnappels et al., 2005).

Two studies found increased TA, GAS, RF, and BF muscle activations of the compensatory limb to control the dynamic body's forward angular momentum after trip perturbations induced by an external obstacle (Pijnappels et al., 2005) and by the split-belt treadmill compared to normal walking (Lee et al., 2019). This study's similar results for the YG and OG indicated that increased TA (125.8% for the YG and 87.99% for the OG), GAS (98.70% for the YG and 78.44% for the OG), RF (225.94% for the YG and 108.49% for the OG), and BF (201.37% for the YG and 106.54% for the OG) muscle activations during the recovery period compared to the pre-perturbation period contributed to restraining the body's forward rotation. Given that joint moments are associated with muscle activations (Zajac and Gordon, 1989; Pijnappels et al., 2005), both the YG and OG showed significantly increased muscle activations and joint moments after split-belt treadmill perturbations with the exception of the dorsiflexion, plantarflexion, and hip extension moments. For insignificantly changes in the dorsiflexion, plantarflexion, and hip extension moments, two possible explanations could be speculated. First, a relatively small sample size may contribute to these results. Second, considering joint moments in the frontal and transverse planes and the sagittal plane contributed to balance recovery after gait perturbations induced by a moveable platform (Liu and Lockhart, 2009), increased TA, GAS, BF muscle activations may in fact contribute to generating ankle and hip joint moments in the frontal and transverse planes.

Previous studies indicated significantly higher lower limb joint moments and muscle responses (e.g., amplitude and activation rate) in young adults after gait perturbations compared to older adults (Tang and Woollacott, 1998; McFadyen and Prince, 2002; Lockhart and Kim, 2006; Liu and Lockhart, 2009). This study indicated that the YG showed higher joint moments after split-belt treadmill perturbations compared to the OG (23.94% higher dorsiflexion moment, 28.84% higher knee flexion moment, 18.46% higher knee extension moment, and 38.86% higher hip flexion moment, respectively). The YG also showed higher muscle activations after split-belt treadmill perturbations than the OG (15.35% higher TA, 72.65% higher RF, and 57.95% higher BF, respectively). Muscle strength in older adults progressively decreased due to sarcopenia (Roubenoff, 2000; Akima et al., 2001), and muscle mass reduced nearly 30–50% caused by the reduction volume and number of muscle fibers with aging (Lexell et al., 1988; Granacher et al., 2008). Considering that aging decreased older adults' muscle strength related to joint moments and muscle activations (Horlings et al., 2008; Pijnappels et al., 2008; Watanabe et al., 2018), decreasing muscle strength may contribute to relatively lower joint moments and muscle activations after split-belt treadmill perturbations. Since resistance training contributed to reversing the age-related loss of muscle strength (Pijnappels et al., 2008), resistance training for lower limb muscles may help to prevent falls.

This study indicated significantly higher CCI of BF/RF after the OG's split-belt treadmill perturbations (18.51% higher than the YG). Previous studies demonstrated higher the CCI of hip muscles (e.g., RF and BF) after slip perturbations induced by a moveable platform or a contaminated floor in older adults compared to young adults (Tang and Woollacott, 1998; Chambers and Cham, 2007). Older adults increase co-contraction of agonist and antagonist muscles to compensate for muscle weakness after a loss of balance (Okada et al., 2001; Chambers and Cham, 2007; Pijnappels et al., 2008; Nagai et al., 2011). Co-contraction increases joint stiffness, which contributes to balance stability by reducing the degree of freedom of the movements of the body segments (Nagai et al., 2011; Nelson-Wong et al., 2012; Schinkel-Ivy and Duncan, 2018). Based on the results of this study, older adults increase the CCI of BF/RF as a compensatory strategy to recover balance after split-belt treadmill perturbations.

This study indicated that the CCI of BF/RF for both groups was significantly lower during the recovery period compared to the pre-perturbation period, unlike a previous study which found a higher CCI of vastus lateralis/hamstring after slip perturbations compared to normal walking. The difference may be due to the increased trunk and COM range of motions after slip perturbations compared to trip perturbations (Lee et al., 2019), and the smaller hip range of motion and limits of stability of the feet in a backward direction than in a forward direction (Humphrey and Hemami, 2010; Lee et al., 2014). This study assumed that slip perturbations may require higher co-contraction by activating both hamstring and vastus lateralis muscles for joint stiffness as a compensation strategy to control for difficult stability challenges. This study indicated that RF muscles related to knee extensions and hip flexions activated more than BF muscles to control the forward rotation of the body after split-belt treadmill perturbations, and may have resulted in a relatively lower CCI during recovery compared to pre-perturbation periods as shown in Figure 2B.

Self-selected comfortable walking speeds were slower than previous studies (~1.15 and 1.05 m/s preferred walking speeds on a treadmill for young adults and older adults, respectively) (Plotnik et al., 2015; Lazzarini and Kataras, 2016). Since no participants received instructions (e.g., how to respond to a perturbation) and there were no practice trials, this study assumed they walked carefully during trials. Previous studies indicated that awareness of upcoming perturbations affected walking performance (e.g., slower walking speeds and shorter step lengths) (Bohm et al., 2015; Okubo et al., 2018).

Our fall-inducing system could be used for train balance-constrained individuals and athletes to improve their responses (e.g., joint moments and muscle responses) after multiple split-belt treadmill perturbations. Compared to fall-inducing systems requiring external mechanisms (Schillings et al., 1996; Pavol et al., 1999; Cham and Redfern, 2002; Okubo et al., 2018), our fall-inducing system using a split-belt treadmill requires less space, offers more precise control of perturbation intensity or increases perturbation intensity gradually, and provides less predictability of gait perturbations with any number of steps before the onset of perturbations. Since perturbation-based gait training reduced and prevented falls in different populations (McCrum et al., 2017), a fall-inducing system using a split-belt treadmill is expected to perturbation-based gait training in clinical or athletic settings.

This study was limited by relatively small sample size. Given that sample size is positively correlated with statistical power (Suresh and Chandrashekara, 2012), a relatively small sample size could limit to generalization of our findings. This study was also limited by gender imbalance in the OG. Joint moments in the frontal and transverse planes, joint stiffness, and the onset detection of muscle activity were not investigated. Future research will increase the sample size, balance gender, and examine joint moments in the frontal and transverse planes, joint stiffness, and the detection of muscle onset. Future research will also investigate the level of physical activity impacts on falls.

## Conclusion

This study characterized the joint moments and muscle responses of the compensatory limb after split-belt treadmill perturbations. Older and younger adults' compensatory limb's joint moments and muscle responses to split-belt treadmill perturbations were compared. Overall, young adults showed higher joint moments and muscle activations during recovery periods after split-belt treadmill perturbations. Older adults showed a higher CCI of BF/RF during recovery periods. This study characterized the joint moments and muscle responses of the compensatory limb after split-belt treadmill perturbations by older adults. Given that gait perturbations encountered during normal walking are a major cause of falls in older adults, the results could teach older adults who train on fall-inducing systems how to compensate for unexpected gait perturbations.

## Data Availability Statement

The original contributions presented in the study are included in the article, further inquiries can be directed to the corresponding author.

## Ethics Statement

The studies involving human participants were reviewed and approved by Institutional Review Boards of the University of Houston. The patients/participants provided their written informed consent to participate in this study.

## Author Contributions

DY collected data, performed data and statistical analysis, interpreted results, and drafted the manuscript. JA assisted with data collection, data analysis, and manuscript preparation. K-HS reviewed the manuscript. B-CL conceived the study, designed the experimental protocols, supervised the research, and edited and reviewed the manuscript. All authors contributed to the article and approved the submitted version.

## Conflict of Interest

The authors declare that the research was conducted in the absence of any commercial or financial relationships that could be construed as a potential conflict of interest.
